# Fluctuation between Fasting and 2-H Postload Glucose State Is Associated with Chronic Kidney Disease in Previously Diagnosed Type 2 Diabetes Patients with HbA1c ≥ 7%

**DOI:** 10.1371/journal.pone.0102941

**Published:** 2014-07-21

**Authors:** Chuan Wang, Jun Song, Zeqiang Ma, Weifang Yang, Chengqiao Li, Xiuping Zhang, Xinguo Hou, Yu Sun, Peng Lin, Kai Liang, Lei Gong, Meijian Wang, Fuqiang Liu, Wenjuan Li, Fei Yan, Junpeng Yang, Lingshu Wang, Meng Tian, Jidong Liu, Ruxing Zhao, Li Chen

**Affiliations:** 1 Department of Endocrinology, Qilu Hospital of Shandong University, Jinan, Shandong, China; 2 China National Heavy Duty Truck Group Corporation Hospital, Jinan, Shandong, China; 3 Lukang Hospital of Jining, Jining, Shandong, China; 4 Department of Endocrinology, Second People's Hospital of Jining, Jining, Shandong, China; 5 Shantui Community Health Center, Jining, Shandong, China; Institute of Endocrinology and Metabolism, Iran, Islamic Republic of

## Abstract

**Objective:**

To investigate how the glucose variability between fasting and a 2-h postload glucose state (2-h postload plasma glucose [2hPG]-fasting plasma glucose [FPG]) is associated with chronic kidney disease (CKD) in middle-aged and elderly Chinese patients previously diagnosed with type 2 diabetes.

**Design and Methods:**

This cross-sectional study included 1054 previously diagnosed type 2 diabetes patients who were 40 years of age and older. First, the subjects were divided into two groups based on a glycated hemoglobin (HbA1c) value of 7%. Each group was divided into two subgroups, with or without CKD. The Chronic Kidney Disease Epidemiology Collaboration (CKD-EPI) equation was used to estimate the glomerular filtration rate (GFR). CKD was defined as eGFR<60 mL/min/1.73 m^2^. Multiple linear regression analysis was used to estimate the association between the 2hPG-FPG and eGFR. The 2hPG-FPG value was divided into four groups increasing in increments of 36 mg/dl (2.0 mmol/L): 0–72, 72–108, 108–144 and ≥144 mg/dl, based on the quartiles of patients with HbA1c levels ≥7%; then, binary logistic regression analysis was used to investigate the association between 2hPG-FPG and the risk of CKD.

**Results:**

In the patients with HbA1c levels ≥7%, the 2hPG-FPG was significantly associated with decreased eGFR and an increased risk of CKD independent of age, gender, body mass index (BMI), systolic blood pressure (BP), diastolic BP, smoking, and drinking, as well as fasting insulin, cholesterol, triglyceride, and HbA1c levels. The patients with 2hPG-FPG values ≥144 mg/dl showed an increased odds ratio (OR) of 2.640 (*P* = 0.033). Additionally, HbA1c was associated with an increased risk of CKD in patients with HbA1c values ≥7%.

**Conclusions:**

The short-term glucose variability expressed by 2hPG-FPG is closely associated with decreased eGFR and an increased risk of CKD in patients with poor glycemic control (HbA1c≥7%).

## Introduction

Chronic kidney disease (CKD), which is characterized by albuminuria or reduced kidney function, is a rapidly increasing global public health problem [1], [2] that significantly increases the risk of cardiovascular events and mortality [3], [4]. Effective treatment for end-stage renal disease (ESRD) is limited to renal replacement therapy, and treatment is predominantly aimed at preventing or slowing disease progression by controlling the risk factors, such as diabetes, hypertension, and dyslipidemia [2], [5], [6].

Among the risk factors, diabetes is the most frequent cause of CKD [5], [7]. Hyperglycemia is closely related to the development of CKD and could be prevented by strict control of blood glucose levels [8]. A growing body of research suggests that glucose variability might accelerate the renal complications of diabetes independently of hyperglycemia; however, the results are inconsistent. One study of type 1 diabetes patients revealed that short-term glucose variability did not predict the development of nephropathy [9]. Two studies indicated that long-term glucose variability could affect CKD in patients with type 2 diabetes [10], [11]. Because of the conflicting results and limited data on the influence of short-term glucose variability on renal function in type 2 diabetes, it is necessary to further clarify this issue.

Monitoring glucose variability is complicated, which greatly limits its application in clinical practice and large epidemiological studies. The most common short-term glucose variability occurs after a meal. It should be determined whether the difference between the fasting and postprandial glucose levels relates to CKD. The calories in a diet differs among subjects because of dietary variations. Thus, the standardization of postprandial glucose levels is greatly limited. The 75-g oral glucose tolerance test (OGTT) is the gold standard test for diagnosing diabetes, and the variability between the fasting plasma glucose (FPG) and the 2-h postload plasma glucose (2hPG) reflects, to some extent, short-term glucose variability. Therefore, a 75-g OGTT was performed for all participants to investigate whether the glucose variability between the FPG and 2hPG states (2hPG-FPG) is associated with CKD.

## Materials and Methods

### Ethics Statement

This work is part of the baseline survey in the REACTION study of the association of diabetes and cancer, which included 259,657 adults (40 years of age and older) in 25 communities across mainland China from 2011 to 2012 [12]. The study was approved by the Ruijin Hospital Ethics Committee of the Shanghai Jiao Tong University School of Medicine. Written informed consent was obtained from the study participants.

### Study population

This study randomly recruited 10,028 subjects (40 years of age and older) in Shandong province from January to April 2012. Based on previous medical history, which was obtained using a standard questionnaire, we selected 1181 patients who were previously diagnosed with type 2 diabetes. The following exclusion criteria were applied: (1) lower 2hPG than FPG; (2) missing data for calculating the eGFR; (3) previously diagnosed kidney disease, including autoimmune or drug-induced kidney disease, nephritis, renal fibrosis or renal failure; (4) previously diagnosed hepatic disease, including fatty liver, liver cirrhosis and autoimmune hepatitis; and (5) any malignant disease. A total of 1054 subjects (604 women) were eligible for the analysis.

### Data collection

A standard questionnaire was used by trained investigators to collect data regarding the demographic characteristics, lifestyle and previous medical histories of the subjects. Body mass index (BMI) was calculated as weight (kg) divided by height squared (m^2^). Blood pressure (BP) was measured 3 consecutive times (OMRON Model HEM-752 FUZZY, Omron Company, Dalian, China) on the left arm after the subjects had remained seated for at least 5 min; the average reading was used for the analysis. After at least 10 h of overnight fasting, venous blood samples were collected between 07∶00 and 09∶00 to measure the FPG, fasting insulin, cholesterol, triglycerides and creatinine levels. The 2hPG was measured after the subjects had completed a 75-g OGTT. HbA1c was measured by high-performance liquid chromatography (VARIANT II and D-10 Systems, BIO-RAD, USA). The homeostasis model assessment of insulin resistance (HOMA-IR) index was calculated as follows: fasting insulin concentration (mIU/L)×FPG concentration (mmol/L)/22.5 [13]. The eGFR was calculated from the creatinine levels using the CKD-EPI formula [14] as shown in Table 1.

**Table 1:** eGFR calculated from the creatinine levels using the CKD-EPI formula.

Sex	Cr (mg/dl)	eGFR (mL/min/1.73 m^2^)
Females	≤0.7	144×(Cr/0.7)^−0.329^×(0.993)^age^
	>0.7	144×(Cr/0.7)^−1.209^×(0.993)^age^
Males	≤0.9	141×(Cr/0.9)^−0.411^×(0.993)^age^
	>0.9	141×(Cr/0.9)^−1.209^×(0.993)^age^

eGFR, estimated glomerular filtration rate; Cr: creatinine.

### Definition

According to the Kidney Disease Outcomes Quality Initiative provided by the US National Kidney Foundation, CKD was defined as eGFR<60 mL/min/1.73 m^2^ [15].

Diabetic patients who were previously diagnosed based on the 1999 World Health Organization (WHO) criteria (FPG≥126 mg/dl [7.0 mmol/L] and/or 2hPG≥200 mg/dl [11.1 mmol/L]) [16] were identified after reviewing their medical histories.

To explore the association between 2hPG-FPG and CKD in diabetic patients with glycemic control variations, we divided the subjects into the following two groups, according to the target HbA1c value (7.0%) [17]: patients with good glycemic control (HbA1c<7%) and patients with poor glycemic control (HbA1c≥7%).

### Statistical analysis

The continuous variables with normal distribution are expressed as the mean ± standard deviation (SD), and the variables with non-normal distribution are presented as the median (interquartile range). The categorical variables are presented as numbers (%). Between-group differences were detected using Student’s *t*-test (the continuous variables in normal distribution), Mann-Whitney U-test (the skewed continuous variables), or chi-squared test (categorical variables). Multiple linear regression analysis was used to estimate the association between 2hPG-FPG and eGFR. The following three models were constructed: Model 1 = not adjusted; Model 2 = adjusted for age, gender, BMI, systolic BP and diastolic BP; Model 3 = Model 2 plus Log (fasting insulin), cholesterol, Log (triglyceride), drinking, smoking and HbA1c. The assumption of a linear relationship between the dependent and independent variables was assessed using a histogram of the residuals, with a scatter plot of the standardized residuals to the standardized predicted values in different models. The 25th, 50th, and 75th percentiles for 2hPG-FPG in the subjects with HbA1c≥7% were 75.6 mg/dl (4.2 mmol/L), 108 mg/dl (6.0 mmol/L) and 144 mg/dl (8.0 mmol/L), respectively. The 2hPG-FPG quartiles in the subjects with HbA1c levels <7% were 50.4 mg/dl (2.8 mmol/L), 84.6 mg/dl (4.7 mmol/L) and 108 mg/dl (6.0 mmol/L). Based on the multiple linear regression analysis, we focused on the subjects with HbA1c levels ≥7% in the following analysis. To facilitate use in clinical practice, the 2hPG-FPG values were divided into four groups increasing in increments of 36 mg/dl (2.0 mmol/L): 0–72, 72–108, 108–144 and ≥144 mg/dl. The association of 2hPG-FPG (the 2hPG-FPG groups were introduced as ordinal dummy variables) with the risk of CKD was estimated using binary logistic regression analysis, and *P*<0.05 was considered to be statistically significant. The data were analyzed using the SPSS 16.0 software (SPSS, Inc., Chicago, IL, USA).

## Results

### Characteristics of study participants

We included 1054 subjects (604 women) who were previously diagnosed with diabetes. The subjects were divided into two groups based on their HbA1c values, using 7% as the cut-off value. Each group was divided into two subgroups, with or without CKD. As shown in Table 2, compared with the non-CKD patients, the CKD patients were more likely to be older, male and smokers in both groups. The CKD patients had higher levels of FPG, 2hPG, 2hPG-FPG and HbA1c in the group with HbA1c values ≥7%.

**Table 2:** Characteristics of the study participants by HbA1c and CKD.

	HbA1c (%) <7 (n = 276)		HbA1c (%) ≥7 (n = 778)	
Characteristics	CKD (n = 25)	non-CKD (n = 251)	CKD (n = 86)	non-CKD (n = 692)
Female (%)	3 (12.0%)	144 (57.4%)**	21 (24.4%)	436 (63.0%)**
Age (years)	70.80±7.48	62.84±9.74**	69.35±8.86	62.62±8.25**
BMI (kg/m^2^)	26.73±3.74	26.80±3.66	26.71±3.23	27.24±3.62
Systolic BP (mmHg)	149.50±22.23	145.45±21.67	149.93±21.12	145.71±21.87
Diastolic BP (mmHg)	76.09±9.61	79.51±10.34	79.89±11.44	80.04±11.74
FPG (mg/dl)	146.61±27.11	137.35±20.13	194.89±64.46	174.13±47.36*
2hPG (mg/dl)	219.67±49.98	218.42±44.30	327.37±84.81	283.66±73.01**
2hPG-FPG (mg/dl)	73.06±44.56	81.08±43.36	132.48±52.02	109.52±49.53**
HbA1c (%)	6.50 (6.10–6.80)	6.50 (6.20–6.80)	8.40 (7.68–9.93)	8.20 (7.50–9.30)*
Fasting insulin (mIU/L)	9.00 (7.05–14.80)	8.90 (6.10–11.90)	9.10 (5.90–1.38)	9.40 (6.48–13.73)
HOMA-IR index	3.33 (2.46–4.67)	2.96 (1.98–4.03)	3.98 (2.70–6.43)	3.77 (2.65–5.90)
Cholesterol (mg/dl)	223.36±44.14	202.36±38.02*	210.41±45.51	215.46±40.08
Triglyceride (mg/dl)	136.44 (91.70–167.90)	129.36 (93.92–183.40)	157.27 (108.98–206.44)	139.10 (100.12–207.32)
Smoking (%)	4 (16.0%)	22 (8.8%)	17 (19.8%)	74 (10.7%)*
Drinking (%)	2 (8.0%)	24 (9.6%)	6 (7.0%)	42 (6.1%)
Creatinine (mg/dl)	1.08 (1.00–1.19)	0.74 (0.68–0.83)**	1.08 (0.99–1.25)	0.75 (0.69–0.82)**
eGFR (mL/min/1.73 m^2^)	52.62 (16.92–58.05)	84.35 (74.45–92.45)**	53.49 (43.57–56.60)	85.26 (75.62–93.09)**

The data are expressed as the means ± SD or numbers (%). CKD, chronic kidney disease; BMI, body mass index; BP, blood pressure; FPG, fasting plasma glucose; 2hPG, 2-h postload plasma glucose; HOMA-IR, homeostasis model assessment of insulin resistance; eGFR, estimated glomerular filtration rate. *P<0.05 vs the CKD group; **P<0.01 vs the CKD group.

### Multiple linear regression analysis

As shown in Table 3, three models were constructed to analyze the association between 2hPG-FPG and eGFR (HOMA-IR and 2hPG-FPG are involved in the calculation of FPG, and we added fasting insulin instead of HOMA-IR to model 3). A linear relationship between the dependent and independent variables in each model was confirmed. A significantly negative association between 2hPG-FPG and eGFR was observed, independent of age, gender, BMI, systolic BP, diastolic BP, smoking, and drinking, as well as fasting insulin, cholesterol, triglyceride, and HbA1c levels only in previously diagnosed diabetes patients with HbA1c values ≥7%. In addition, a close relationship between HbA1c and decreased eGFR levels was observed in both groups.

**Table 3:** Multiple linear regression analysis of the relationship between 2hPG-FPG and eGFR.

		HbA1c (%) <7		HbA1c (%) ≥7	
Models	Independent variable	β Coefficient (95% CI)	*P*-value	β Coefficient (95% CI)	*P*-value
Model 1	2hPG-FPG, per mg/dl	−0.018 (−0.062 to 0.026)	0.417	−0.042 (−0.064 to −0.020)	**<0.001**
Model 2	2hPG-FPG, per mg/dl	0.013 (−0.018 to 0.043)	0.417	−0.035 (−0.053 to −0.017)	**<0.001**
Model 3	2hPG-FPG, per mg/dl	0.011 (−0.020 to 0.041)	0.492	−0.023 (−0.041 to −0.004)	**0.018**
	Log (HbA_1c_), per unit	−46.082 (−90.153 to −2.012)	**0.040**	−23.194 (-35.654 to −10.733)	**<0.001**

Model 1: not adjusted; Model 2: adjusted for age, gender, BMI, systolic BP and diastolic BP; Model 3: Model 2 plus Log (fasting insulin), cholesterol, Log (triglyceride), drinking and smoking.

### Binary logistic regression analysis

The subjects were divided into four groups based on their 2hPG-FPG values, in increasing increments of 36 mg/dl (2.0 mmol/L), as follows: 0–72, 72–108, 108–144 and ≥144 mg/dl. As shown in Table 4, we analyzed the association between the increased 2hPG-FPG levels and the risk of CKD in three models. As expected, 2hPG-FPG could significantly increase the risk of CKD in patients with HbA1c values ≥7%; but not in subjects with HbA1c<7%. In model 1, the patients with 2hPG-FPG values ranging from 108–144 and ≥144 mg/dl had a significantly increased risk of CKD (odds ratio [OR] = 2.339 and 3.298, respectively). After adjusting for age, gender, BMI, systolic BP and diastolic BP, these two groups also presented increased ORs (2.397 and 3.662, respectively). With further adjusting for fasting insulin, cholesterol, triglycerides, smoking, drinking and HbA1c, the patients with a 2hPG-FPG value ≥144 mg/dl showed an increased risk of CKD (OR = 2.640, *P* = 0.033) Additionally, HbA1c was associated with an increased risk of CKD in patients with HbA1c levels ≥7%.

**Table 4:** Binary logistic regression analysis of the relationship between 2hPG-FPG and CKD.

		HbA1c (%) <7		HbA1c (%) ≥7	
Models	Independent variable	Odds ratio (95% CI)	*P*-value	Odds ratio (95% CI)	*P*-value
Model 1	2hPG-FPG, mg/dl				
	Group 1 (0–72)	1 (reference)		1 (reference)	
	Group 2 (72–108)	1.292 (0.509–3.279)	0.589	1.385 (0.611–3.138)	0.435
	Group 3 (108–144)	0.762 (0.232–2.508)	0.655	2.339 (1.094–4.999)	**0.028**
	Group 4 (≥144)	0.472 (0.058–3.852)	0.483	3.298 (1.577–6.895)	**0.002**
Model 2	2hPG-FPG, mg/dl				
	Group 1 (0–72)	1 (reference)		1 (reference)	
	Group 2 (72–108)	0.862 (0.266–2.788)	0.804	1.448 (0.589–3.558)	0.420
	Group 3 (108–144)	0.364 (0.088–1.511)	0.164	2.397 (1.038–5.534)	**0.041**
	Group 4 (≥144)	0.200 (0.017–2.325)	0.198	3.662 (1.575–8.514)	**0.003**
Model 3	2hPG-FPG, mg/dl				
	Group 1 (0–72)	1 (reference)		1 (reference)	
	Group 2 (72–108)	0.812 (0.233–2.832)	0.743	1.220 (0.477–3.120)	0.678
	Group 3 (108–144)	0.330 (0.068–1.606)	0.170	2.076 (0.874–4.929)	0.098
	Group 4 (≥144)	0.240 (0.020–2.878)	0.260	2.640 (1.083–6.436)	**0.033**
	HbA_1c_, per % unit	0.717 (0.232–2.222)	0.565	1.295 (1.096–1.529)	**0.002**

Model 1: not adjusted; Model 2: adjusted for age, gender, BMI, systolic BP and diastolic BP; Model 3: Model 2 plus Log (fasting insulin), cholesterol, Log (triglyceride), drinking and smoking.

## Discussion

There has been no consensus to define glucose variability, although the concept is intuitively comprehensible. The most common understanding of glucose variability refers to the within-day glucose variability, which is evaluated using the blood glucose values obtained by self-monitoring or continuous glucose monitoring (CGM) [18]. The other predominant concepts are between-day fasting glycemia variability, postprandial glycemic peaks, variability of HbA1c over time, and hypoglycemic episodes [18]. Numerous indices are calculated to estimate glucose variability [18]. Regardless of which index is used, continuous glucose monitoring within one day or longer is required, which complicates and greatly limits its application in clinical practice and large epidemiological studies. Because the most common glucose variability occurs after a meal and the definition of glucose variability is uncertain, the difference between fasting and postprandial glucose levels might reflect the state of glucose variability (to some extent). Moreover, to standardize the calories intake in the meal, 75 g OGTT was done for all subjects and 2hPG was used to express postprandial glucose. Therefore, in this study, glucose variability is expressed by the 2hPG-FPG.

Although the effect of glucose variability on the development of complications of diabetes has been postulated and discussed in recent years [19], [20], [21], [22], the study results are controversial. Kilpatrick et al. analyzed Diabetes Control and Complications Trial (DCCT) patients with type 1 diabetes and observed that the within-day glucose variability (calculated using the SD and mean amplitude of glycemic excursions) in the DCCT did not predict the development of retinopathy or nephropathy [9]. Two recent studies revealed that HbA1c variability (expressed by the SD of HbA1c) independently affected the development of CKD in type 2 diabetes patients [10], [11]. The conflicting results might be attributed to the different study populations and various indices used to express glucose variability in the various studies. The latter two studies [10], [11] observed the effect of long-term glycemic variability (the SD of HbA1c) on renal complications. Whether short-term glucose variability affects change in renal function in type 2 diabetes remains to be explored. To verify this hypothesis, we investigated the association between short-term glucose variability (expressed by 2hPG-FPG) and the risk of CKD in type 2 diabetes. To analyze this association, we divided the subjects into two groups based on their glycemic control, with 7% HbA1c as the cut-off value. In diabetes patients with good glycemic control (HbA1c<7%), 2hPG-FPG and HbA1c levels were not associated with the risk of CKD, indicating that, with good glycemic control, short-term glucose variability would not significantly affect the development of CKD. The small sample of CKD in the group with HbA1c<7% might influence the accuracy of the results. Additional studies with a larger sample size of CKD patients should be performed to verify this observation. In the subjects with poor glycemic control (HbA1c≥7%), both 2hPG-FPG and HbA1c levels were significantly associated with decreased eGFR and an increased risk of CKD, which suggested that, with poor glycemic control, both the average blood glucose and the short-term glucose variability contributed to the development of CKD. The underlying mechanism for this phenomenon might be found in diabetic rodent studies. One study found that blood glucose fluctuation could accelerate the trend of kidney fibrosis through the ERK/MAPK and TGF-β/Smad signaling pathways in diabetic mice by increasing collagen production and inhibiting collagen degradation [23]. Another study observed that diabetic rodents with higher blood glucose fluctuation always showed a more serious degree of glomerular sclerosis compared with that shown in rodents with sustained high blood glucose levels [24].

In addition to hyperglycemia, the traditional risk factors of CKD include sex, age, obesity, hypertension, smoking, drinking, dyslipidemia and insulin resistance [2], [6]. We adjusted for age, gender, BMI, systolic BP, diastolic BP, smoking, and drinking, as well as fasting insulin, cholesterol, triglycerides, and HbA1c levels, to ensure that our results were more reliable. Because HOMA-IR and 2hPG-FPG included FBG in the calculation, we added fasting insulin instead of HOMA-IR to model 3. After adjusting for the above risk factors, 2hPG-FPG was significantly associated with decreased eGFR and an increased risk of CKD in patients with HbA1c levels ≥7%; there was not a significant association regarding patients with HbA1c levels <7%.

Our study has some limitations. First, a cross-sectional study could not infer causality between 2hPG-FPG and CKD. Second, the fluctuation between a fasting and postprandial glucose state (2hPG-FPG) could not precisely reflect the glucose fluctuation. As a simple indicator of glucose fluctuation, 2hPG-FPG might be easily applicable to clinical practice. Third, our study included only middle-aged and elderly Chinese subjects; therefore, these results might not be applicable to subjects of different ages or ethnicities. Fourth, the sample size of CKD patients in the group with HbA1c levels <7% was limited, which would lead to an erroneous estimation of the effect of 2hPG-FPG on the risk of CKD. Fifth, the duration of diabetes was unknown; a longer disease duration could increase the risk of CKD. Additionally, other risk factors for CKD, such as uric acid, were not included in this study, which might affect the results. Finally, without measuring protein in the urine, the GFR based on creatinine and estimated by the CKD-EPI equation might not accurately reflect the kidney function, which might influence the accurate estimation of CKD. The gold standard method for measuring the GFR (isotope clearance measurement) is expensive and time-consuming, and the use of creatinine-based equations to estimate the GFR is reasonable for large epidemiological studies. The CKD-EPI equation is more accurate and precise than the MDRD study equation and the Cockcroft-Gault equation, particularly for the subjects with eGFR levels <60 mL/min/1.73 m^2^ [25]. The CKD-EPI equation might be an optimum estimation of the GFR.

We found that 2hPG-FPG was closely associated with decreased eGFR and an increased risk of CKD in previously diagnosed type 2 diabetes patients with HbA1c levels ≥7%; it was not associated in patients with HbA1c levels <7%. In patients with poor glycemic control, the average blood glucose (expressed by HbA1c) and the short-term glucose variability (expressed by 2hPG-FPG) could increase the risk of CKD. With blood glucose control within the target HbA1c value (7.0%), the short-term glucose variability might not continue to affect the change in renal function, which indicates the fundamental role of good control of the average blood glucose in CKD prevention. Additional longitudinal studies are needed to verify these study results and to explore the degree to which the 2hPG-FPG value should be controlled in clinical practice to benefit CKD patients.
